# Risk Factors for Prolonged Opioid Use Following Total Hip Arthroplasty and Total Knee Arthroplasty: A Narrative Review of Recent Literature

**DOI:** 10.1177/10600280221133078

**Published:** 2022-10-31

**Authors:** Carter VanIderstine, Emily Johnston

**Affiliations:** 1 College of Pharmacy, Dalhousie University, Halifax, Nova Scotia, Canada; 2 Pharmacy Department Halifax Infirmary, Nova Scotia Health, Halifax, Nova Scotia, Canada

**Keywords:** analgesia, opioids, orthopedics, prescribing patterns, risk management

## Abstract

**Objective:**

To provide pharmacists and other health care professionals with the knowledge required to minimize the risk of prolonged opioid use following total hip arthroplasty (THA) and total knee arthroplasty (TKA).

**Data Sources:**

A literature search of PubMed and Embase was performed, and included the search terms: (opioid OR opiate OR opium) AND (risk factor OR predict*) AND (arthroplasty OR replacement) NOT shoulder.

**Study Selection and Data Extraction:**

Randomized control trials, cohort studies (both prospective and retrospective), systematic reviews, and meta-analyses were included if risk ratios (RRs) or odds ratios (ORs) were reported and published within the last 5 years.

**Data Synthesis:**

]Twenty studies met inclusion criteria, including 2 meta-analyses and 2 prospective studies. There were several risk factors that overlapped between studies and presented clinically significant risks for prolonged opioid use following THA and TKA surgery. Of these, age < 65 (RRs: 1.15-9.36), preoperative opioid use (RRs: 1.09-7.81), larger quantities of opioids prescribed at discharge (RRs: 1.26-8.81), and TKA surgery (RRs: 1.73-6.07) were the most significant. Several risk factors were recently described, including migraines (RRs: 1.14-5.11) and fibromyalgia (RRs: 1.1-2.3) that may be of interest for further research.

**Relevance to Patient Care and Clinical Practice:**

This review presents a discussion of the factors associated with prolonged opioid use following THA and TKA surgeries, which are among the most common orthopedic surgeries.

**Conclusions:**

Prescribers should carefully consider patient-specific factors when prescribing opioids as there are several factors, including age, surgery type, and medical conditions that can predispose patients to prolonged opioid use.

## Introduction

The 2 most frequently performed orthopedic surgeries are total hip arthroplasty (THA) and total knee arthroplasty (TKA). In both of these cases, adequate pain management is crucial in a patient’s recovery and management of postoperative complications.^1,2^ These types of surgery are used to treat pain associated with arthritis, improve mobility, and most importantly, improve patients’ overall quality of life.^3^ Over the next 10 years, it is estimated that the number of THA and TKA surgeries in the United States alone will increase by 70% to 85%.^4^

Recently, the American Academy of Orthopaedic Surgeons (AAOSs) released guidelines for opioid prescribing, which recommend that THA and TKA patients receive no more than 400 Morphine Milligram Equivalents (MMEs); however, no recommendation was made as to the duration of these prescriptions.^5^ In clinical practice, however, patients are being prescribed in some cases double this recommendation.^6^ The consequences of this include a higher likelihood of diversion, increased risk to children in the household, and increased risk of inappropriate use.^7,8^ As it has been shown that the majority of patients do not know how to properly dispose of their medication, there is growing interest in identifying which patients are at the highest risk of prolonged opioid use so that the prescription can be adjusted accordingly.^6,7^

The most recent estimate is that upward of 10% of opioid-naïve patients will develop a dependence postoperatively, with a higher percentage of preoperative opioid users continuing long term, regardless of surgery type.^8,9^ Therefore, this narrative review will explore risk factors for prolonged opioid use following THA and TKA. The variety of definitions of prolonged postoperative opioid use reported in the literature will also be explored. In contrast to previously published meta-analyses, this narrative review aims to find connections between a wider range of study designs to help pharmacists better navigate a patient’s specific opioid requirements postoperatively and provide more appropriate opioid stewardship in elective arthroplasty programs.

## Methods

Relevant studies were identified from PubMed, Medline, and Embase. The search terms and strategies for each database used in this narrative review included: (opioid OR opiate OR opium) AND (risk factor OR predict*) AND (arthroplasty OR replacement) NOT shoulder. Randomized control trials, systematic reviews, cohort studies, and meta-analyses published between January 2016 and January 2022 were considered for inclusion if risk ratios (RRs) or odds ratios (ORs) were reported. Searches were originally performed in July 2021 and performed again prior to submission. As this is an ever-evolving field of research, 5 years was chosen to get the most up-to-date information. Given that this study only examined hip and knee arthroplasty, all others were excluded from the database search terms. To better examine risk factors, both prospective and retrospective studies were included in this study and were not specific to North America. For the purpose of this study, “prolonged opioid use” was defined as opioid use lasting beyond the expected acute postoperative pain response, which current literature suggests is ≤ 14 days for TKA and THA surgeries.^10^ This broad definition was chosen to capture and characterize the wide range of interpretations of prolonged opioid use reported in the literature. Furthermore, studies that defined prolonged opioid use as beginning more than a year after surgery were excluded due to the potential for confounding variables that could occur in that time.

## Data Synthesis

### Study Demographics

Since 2016, 20 studies have been published on the risk factors for prolonged opioid use following TKA or THA surgery: 2 of which were conducted prospectively, 2 were meta-analyses, and the remainder were conducted retrospectively.^11-30^ Importantly, both prospective studies were carried out in the United States, had overlapping eligibility periods, and tracked patients over the course of a year. However, they were performed in different parts of the country and had differing exclusion criteria. As for the meta-analyses, both were published in 2020, but again, the inclusion criteria differed, potentially leading to variability when comparing studies. One of the meta-analyses included studies which defined prolonged opioid use as being fewer than 3 months, whereas, the other did not.^21,22^ Furthermore, one of the meta-analyses pooled risk factors following TKA and THA into one analysis, whereas the other conducted separate analyses stratified by surgery.

### Patient Factors in the Literature

In summary, risk factors for prolonged opioid use were studied in patients having received TKA and/or THA surgery between 1999 and 2019. A total of 10 risk factors were identified: gender, age, preoperative opioid use, discharge prescription size, TKA, anxiety/ depression, benzodiazepine (BZD) use, alcohol and substance use, migraines, and fibromyalgia. As for surgery type, 8 studies included solely TKA patients,^11,14,16-18,27-30^ 4 included solely THA patients,^13,19,25,26^ and 7 included both TKA and THA patients.^12,15,20-24^ Furthermore, 3 studies reported risk factors over time.^19,26,27^ The number of participants included in each study ranged from 338 to 416 321 with average participants’ age falling between 59.7 and 80. The proportion of female participants varied from 7% to 63.8%, as did the proportion of white participants at 35.8% to 95%. One limitation of the current study is that the definition of “prolonged opioid use” varied among the studies included ranging from 2 weeks to 2 years with 6 continuous months being the most used description. Risk factors discussed include those where the RR for each risk factor was reported in at least 4 studies to ensure a consensus could be achieved. Study demographics are summarized in Tables 1 and 2. RRs and ORs discussed here can be found in Table 3.

**Table 1.** Study Designs of Studies Included in This Review.

PMID	Author	Study period	Location	Study type	Surgery type	No. of participants
26871536	Goesling et al^20^	2010-2013	USA	Prospective	TKA, THA	567
27130165	Inacio et al^13^	2001-2012	AUS	Retrospective	THA	9525
28413136	Bedard et al (2017)^27^	2007-2015	USA	Retrospective	TKA	73 959
28917616	Bedard et al (2017)^26^	2007-2015	USA	Retrospective	THA	37 393
28887020	Cancienne et al^14^	2007-2016	USA	Retrospective	TKA	113 337
28927564	Hadlandsmyth et al^30^	2013-2015	USA	Retrospective	TKA	6653
29676839	Jorgensen et al (2018)^15^	2011-2013	DEN	Retrospective	TKA, THA	8975
28844770	Kim et al^16^	2016	USA	Retrospective	TKA	338
29753617	Namba et al^17^	2008-2011	USA	Retrospective	TKA	23 726
29198871	Politzer et al^29^	2007-2013	USA	Retrospective	TKA	66 950
31567804	Prentice et al^19^	2008-2011	USA	Prospective	THA	12 560
32307293	Consson et al^11^	2016-2017	USA	Retrospective	TKA	621
32389404	Delaney et al^25^	2013-2016	USA	Retrospective	THA	1403
33006460	Lavoie-Gagne et al^21^	1999-2019		Meta-analysis	TKA	164 998
33006460	Lavoie-Gagne et al^21^	1999-2019		Meta-analysis	THA	33 405
32839063	Stambough et al^12^	2018-2019	USA	Retrospective	TKA, THA	425
32468169	Wu et al^22^	2015-2019		Meta-analysis	TKA, THA	416 321
33484147	Chaudhary et al^24^	2006-2014	USA	Retrospective	TKA, THA	29 767
33074953	Ruddell et al^23^	2016	USA	Retrospective	TKA, THA	507
33141227	Wilt et al^28^	2017	USA	Retrospective	TKA	837
35382897	Ward et al^18^	2014-2017	USA	Retrospective	TKA	1507

Abbreviations: AUS, Australia; DEN, Denmark; PMID, PubMed identifier; THA, total hip arthroplasty; TKA, total knee arthroplasty; USA, United States.

**Table 2.** Patient Demographics of Studies Included in This Review.

PMID	Author	Mean age (years)	% female	% white	Definition of prolonged use
26871536	Goesling et al^20^	63	60	95	6 months
27130165	Inacio et al^13^	80	51.3		3 months
28413136	Bedard et al (2017)^27^		63.8		1 month
28917616	Bedard et al (2017)^26^		41		1 months
28887020	Cancienne et al^14^		63.5		3 months
28927564	Hadlandsmyth et al^30^	66	7	78	12 months
29676839	Jorgensen et al (2018)^15^	67.7	57.9		9 months
28844770	Kim et al^16^	62.4	57		6 months
29753617	Namba et al^17^	68	62.9	65.9	12 months
29198871	Politzer et al^29^		63.4		6 months
31567804	Prentice et al^19^	67	58.5	78.6	90 days
32307293	Consson et al^11^	62.3	57.6	92.3	15 days
32389404	Delaney et al^25^		57.3	94	3 months
33006460	Lavoie-Gagne et al^21^				1-24 months
33006460	Lavoie-Gagne et al^21^				
32839063	Stambough et al^12^	63.8	57	79	1 refill
32468169	Wu et al^22^				2 months
33484147	Chaudhary et al^24^	55.9	52.6	52.7	6 months
33074953	Ruddell et al^23^	66.6	63.1	79	1 month
33141227	Wilt et al^28^				6 months
35382897	Ward et al^18^	59.7	81.1	35.8	3 months

Abbreviation: PMID, PubMed identifier.

**Table 3.** Risk Factor Risk Ratios (RRs) for Prolonged Opioid Use Following Hip and Knee Replacement Surgery.

PMID	Author	Female sex	Age <65	TKA surgery	
26871536	Goesling et al^20^	0.98 [0.49, 1.91]	1.00 [0.97, 1.04]	**3.36 [1.04, 14.6]**	
27130165	Inacio et al^13^	**1.4 [1.00, 1.96]**			
28413136	Bedard et al (2017)^27^	**1**-**2**	**1.1-1.7**		
28917616	Bedard et al (2017)^26^	0.9**-**1.1	**1.4-2.1**		
28887020	Cancienne et al^14^	0.97 [0.93, 1.00]	**1.82 [1.63, 2.04]**		
28927564	Hadlandsmyth et al^30^	0.53 [0.21, 1.35]	1.30 [0.83, 2.04]		
29676839	Jorgensen et al (2018)^15^			**1.73 [1.49, 2.01]**	
28844770	Kim et al^16^	0.45 [0.15, 1.4]	4.55 [0.47, 50]		
29753617	Namba et al^17^	1.01**-**1.07	**1.09-1.17**		
29198871	Politzer et al^29^	**1.23 [1.2, 1.25]**	**1.42 [1.4, 1.46]**		
31567804	Prentice et al^19^	**1-1.17**	**1.03-1.15**		
32307293	Consson et al^11^	1.54 [0.93, 2.57]	***P* < 0.0001**		
32389404	Delaney et al^25^	1.1 [0.71, 1.72]			
33006460	Lavoie-Gagne et al^21^ (TKA)	0.53**-**1.9	**9.36**		
33006460	Lavoie-Gagne et al^21^ (THA)	0.9**-**2.4	0.7**-**2.1		
32839063	Stambough et al^12^	1.13 [0.69, 1.76]	**1.52 [0.81, 2.86]**	**2.68 [1.61, 4.46]**	
32468169	Wu et al^22^	**1.11 [1.04, 1.19]**	**1.27 [1.16, 1.39]**		
33484147	Chaudhary et al^24^	**1.11 [1.05, 1.18]**			
33074953	Ruddell et al^23^	1.4 [0.87, 2.25]		**6.07 [3.29, 11.2]**	
33141227	Wilt et al^28^	0.84**-**1.2	**1.05**		
35382897	Ward et al^18^	1.02 [0.94, 1.10]			
PMID	Author	Preoperative opioid use	Larger discharge Rx	BZD use	Alcohol use
26871536	Goesling et al^20^	**3.9 [4.59, 18.74]**			
27130165	Inacio et al^13^	**3.63-5.18**		**1.56 [1.13, 2.16]**	2.16 [0.75, 6.22]
28413136	Bedard et al (2017)^27^	1.6**-**11.1			**1.1-2.0**
28917616	Bedard et al (2017)^26^	1.7**-**11.1			**1.2-2.2**
28887020	Cancienne et al^14^	**5.47 [5.31, 5.64]**			**1.19 [1.10, 1.29]**
28927564	Hadlandsmyth et al^30^	**7.81 [4.07, 15.00]**		1.1 [0.69, 1.75]	**1.74 [1.01, 2.99]**
29676839	Jorgensen et al (2018)^15^	**1.46 [1.24, 1.73]**			
28844770	Kim et al^16^	5.97 [0.49, 72.23]			
29753617	Namba et al^17^	**1.04-1.09**			**1.03-1.28**
29198871	Politzer et al^29^	**3.75 [3.59, 3.93]**			
31567804	Prentice et al^19^	**1.06-1.11**			**1.07-1.1**
32307293	Consson et al^11^		**8.81 [6.24, 12.45]**		
32389404	Delaney et al^25^		**2.28 [1.22, 4.26]**		
33006460	Lavoie-Gagne et al^21^ (TKA)	**3.75-3.75**	0.85**-**10.2		**1.1-1.5**
33006460	Lavoie-Gagne et al^21^ (THA)	**3.63-3.63**		**2.52-2.52**	**1.2-2.2**
32839063	Stambough et al^12^	***P* < 0.0001**		**1.44 [0.33, 6.30]**	
32468169	Wu et al^22^	**1.73 [1.63, 1.84]**		**1.38 [1.26, 1.51]**	1.57 [0.96, 2.55]
33484147	Chaudhary et al^24^	**1.53-3.03**			
33074953	Ruddell et al^23^	**7.59 [4.24, 13.56]**	**1.26 [1.0, 1.6]**	0.92 [0.42, 2.02]	
33141227	Wilt et al^28^	**1.49-1.7**		1.26**-**1.43	
35382897	Ward et al^18^	**1.60 [1.45, 1.78]**		1.01 [0.94, 1.08]	
PMID	Author	Anxiety	Depression	Migraines	Fibromyalgia
27130165	Inacio et al^13^	1.0 [0.66, 1.5]	**1.7 [1.2, 2.41]**	**5.11 [1.08, 24.18]**	
28413136	Bedard et al (2017)^27^	**1.1 [1.03, 1.1]-2.2 [2.0, 2.2]**			**1.1-1.9**
28917616	Bedard et al (2017)^26^	**1.1 [1.1, 1.1]-2.2 [2.1, 2.4]**			1**-**1.9
28887020	Cancienne et al^14^		**1.32 [1.28, 1.36]**	**1.22 [1.16, 1.29]**	**1.17 [1.13, 1.21]**
28927564	Hadlandsmyth et al^30^		1.04 [0.63, 1.71]		
29676839	Jorgensen et al (2018)^15^	**1.38 [1.15, 1.66]**			
28844770	Kim et al^16^	1.53 [0.21, 5.29]			
29753617	Namba et al^17^	**1.05-1.11**	**1.02-1.2**		**1.03-1.15**
31567804	Prentice et al^19^	**1-1.2**	0.99**-**1.03		0.98**-**1.18
32389404	Delaney et al^25^	1.36 [0.49, 3.8]	1.25 [0.40, 3.97]		
33006460	Lavoie-Gagne et al^21^ (TKA)	**1.5**		**1.22-1.22**	**1-2.3**
33006460	Lavoie-Gagne et al^21^ (THA)	**1.1-2.2**		**1.76-5.11**	**1-1.9**
32468169	Wu et al^22^	**1.11 [1.08, 1.15]**	**1.12 [1.02, 1.23]**	**1.14 [1.03, 1.26]**	**1.35 [1.13, 1.62]**
33484147	Chaudhary et al^24^	**1.16 [1.06, 1.27]**	**1.15 [1.08, 1.22]**		

Studies with no data on these risk factors were excluded from the table. Bolded values were statistically significant. Brackets represent 95% confidence intervals.

Abbreviations: BZD = benzodiazepine; PMID, PubMed identifier; THA, total hip arthroplasty; TKA, total knee arthroplasty.

### Gender

As most arthroplasty patients are female, one of the most frequently assessed risk factors was that of gender, where all but one study reported the RR of female gender for prolonged opioid use following THA or TKA surgery. The study that did not report an RR did, however, report that females were statistically more likely to develop prolonged opioid use compared with men (*P* = 0.002).^15^ Of the studies that did report an RR for female gender, the ratio only reached statistical significance in 6 studies where they varied from 1.1 to 1.4.^13,19,22,24,27,29^ The most protective RR was found in a study by Kim et al^16^ among TKA patients, who found a statistically non-significant RR of 0.45 (CI: 0.15, 1.4). The wide confidence interval suggests this study was not appropriately powered to achieve this difference with *n* = 338. The largest RR came from a meta-analysis of THA patients by Lavoie-Gagne et al^21^ who reported it to be between 0.9 and 2.4 (confidence intervals not reported), although again not statistically significant. Of the 4 studies that examined risk factors over the course of a year, 2 achieved statistical significance.^19,26,27^ Bedard et al^27^ found a peak RR of 2 at 3 months postoperative while Prentice et al^19^ reported their highest RR of 1.17 at the 271 to 360 days postoperative timepoint. When all the studies were further stratified by surgery type, there did not appear to be any difference in the RR of female gender between THA and TKA surgeries, suggesting that if female gender were a risk factor for prolonged opioid use, it is likely independent on the type of surgery.

Taken together, only 1 in 3 studies reported that women were at a significantly higher risk of prolonged opioid use. On top of this, the results were quite variable and only demonstrated a modest increase in risk, therefore, it is unlikely that women are more likely to exhibit a clinically relevant increase in opioid usage following THA or TKA surgery compared with men.

### Age

As previously mentioned, most patients undergoing THA and TKA surgeries are over the age of 60, making age a valuable risk factor to assess. Because the average age of participants fell between 60 and 80, many studies chose to use either 65 or 50 as a cut-off to have enough patients above and below the cut-off to assess how age may impact a person’s risk of prolonged opioid use. Of the 20 studies we included, 15 assessed the effect of age on opioid use and of those, the RR for younger individuals ranged from 0.7 to 9.36 and reached statistical significance in 11 studies, with only one study reporting an RR less than 1.^11,12,14,17,19,21,22,26-29^ It is also notable that the RR of 9.36 was found in a very specific subset of TKA patients between the ages of 31 and 49.^21^ In the Lavoie-Gagne et al^21^ study, risk factors were stratified by surgery type and authors reported an RR of 9.36 for TKA patients and 0.7 to 2.1 for THA patients, with the former being statistically significant. This trend was also seen when comparing other studies as well, where TKA patients under the age of 65 tended to show higher RRs compared with THA patients under the age of 65.

When assessed over the course of a year, Bedard et al (2017) showed that the RR for older patients increased over time for both THA and TKA patients, whereas Prentice et al^19^ and Namba et al^17^ reported the highest RR within the first 90 days following surgery and decreasing thereafter.^26,27^ One possible reason for this difference may be due study design which drew information from the different insurance companies used by each of these groups. They had overlapping enrollment periods and were all performed in the United States; however, the Bedard et al studies analyzed data from Humana, which only covers commercial and Veterans’ policies, while the Prentice and Namba studies used Kaiser Permanente data, which primarily includes personal and family plans, suggesting there may be underlying differences in the demographics of their studies. There is an important consideration when interpreting these results: the age cut-off for determining risk tended to vary with some studies choosing 50 years of age, others chose 65, and some examined trends in 10-year increments, which is where some of the variability in RR originates.

In total: (1) 3 of 4 studies showed that as arthroplasty patients get older, their risk of prolonged opioid use decreases significantly; and (2) on average, patients under the age of 65 are more likely to use opioids long term following THA or TKA surgery than older patients, an important factor to keep in mind when prescribing for these patients although no mechanism has been hypothesized for this difference.

### Preoperative Opioid Use

Given that most patients undergoing THA or TKA surgery are prescribed opioids for postoperative pain, it is important to note whether these patients have taken them in the past. All but one study assessed preoperative opioid use as a potential risk factor for prolonged use and of those, 15 reported statistically significant results. RRs ranged from 1.04 to 7.81.^12-24,26-30^ When tracked over the course of a year, 4 studies found the highest RR at 8 months postoperative and all 3 of the Lavoie and Bedard studies reported similar RRs between THA and TKA patients.^17,19,21,26,27^ Bedard et al^26^,^27^ further showed that while 28% to 33% of preoperative opioid users were still consuming opioids 1 year after surgery, only 2.9% to 3.3% of opioid-naïve patients were. The results reported by Hadlandsmyth et al^30^ were even more dramatic, wherein 57% of chronic preoperative opioid users were consuming opioids 1 year after surgery, while just 2% of opioid-naïve patients were.

In summary, most of these studies demonstrated that preoperative opioid use presents both a statistically relevant and clinically important factor to consider when prescribing opioids to THA and TKA patients. In terms of risk stratification, the more frequently a patient uses opioids prior to surgery, the higher their risk of prolonged use. Even one fill within the 3 months prior to surgery can put them at risk of prolonged use postoperatively.^31,32^

### Discharge Prescription

In this research, we found 5 studies which reported the average MMEs that patients were prescribed at discharge and only 4 that considered it a potential risk factor.^11,12,21,23,25^ In these studies, patients were given between 154 and 519 MMEs at discharge, although these numbers varied greatly with standard deviations upward of 308 MMEs.^11,21,23,25^ As previously mentioned, the AAOS recommends no more than 400 MMEs are prescribed for postoperative THA and TKA patients. Meaning that in half of the studies where discharge MMEs were reported, patients were receiving more than the recommended amount.^5^ Furthermore, of the 4 studies assessing larger discharge prescriptions as a risk factor, 3 were statistically significant and showed RRs ranging from 1.26 to 8.81. This may be a risk factor because studies have shown that the majority of patients do not know how to properly dispose of their medication, making it available for them to use for other purposes, where opioids may not necessarily be indicated.^33^

While increasing the amount of opioids (MMEs) prescribed at discharge appears to play a significant role as a risk factor for prolonged opioid use, more work needs to be done to elucidate the exact magnitude of this effect. In recent years, there have been a growing number of opioid sparing protocols developed that have been able to reduce the MMEs given to patients by up to 50% to 75% without having a negative impact on patient-reported pain and helping to reduce the amount of left-over medication that can be used for other purposes, such as non-surgical pain where opioids may not be indicated.^34-36^ These studies have also shown that opioid sparing protocols reduce the number of refills and leftover tablets, leading to reduced workload for community pharmacists since opioids are controlled medications and therefore require special dispensing and disposal protocols.

### Total Knee Arthroplasty

Four of the studies included here directly compared THA versus TKA as a potential risk factor for prolonged opioid use, all of which were statistically significant. RRs for TKA surgery ranged from 1.73 to 6.07 and what is notable about these 4 studies is that they share very few similarities.^12,15,20,23^ For example, each study uses a different definition of prolonged opioid use, 1 study was completed prospectively,^20^ 1 study was performed in Denmark,^15^ and enrollment periods for the 3 performed in the United States did not overlap.^12,20,23^ Interestingly, despite reporting that TKA patients were at an ~6-fold increased risk of prolonged opioid use, Ruddell et al^23^ reported that TKA patients received fewer MMEs at discharge than THA patients (490.8 ± 292.5 vs 527 ± 332). None of these studies provided a hypothesis as to why TKA patients are at an increased risk. It is possible that this increased risk is due to the presence of other risk factors among these patients, however, further studies are required in this area. Alternative methods of analgesia discussed in these articles included peripheral nerve blocks, local analgesia, and cannabinoids, all of which have been shown to be efficacious in TKA patients.^37-39^

### Anxiety/ Depression

In total, 13 studies reported the RR for anxiety and/or depression as a potential risk factor for prolonged opioid use. In 5 stu-dies, anxiety and depression were grouped together,^15,16,21,26,27^ 6 studies measured them separately,^13,17,19,22,24,25^ and 2 measured an RR for depression, but not anxiety.^14,30^ When the 2 conditions were grouped together, 4 of the 5 studies^15,21,26,27^ showed statistically significant RRs ranging from 1.1 to 2.2. When analyzed separately, 4 of 6 studies^17,19,22,24^ found statistically significant RRs for anxiety alone ranging from 1.05 to 1.2 and 5 studies^13,14,17,22,24^ reported statistically significant RRs for depression ranging from 1.12 to 1.7. The RRs for both anxiety and depression tended to increase over time.^17,19,26,27^ Given that patient’s medical records do not always document anxiety or depression as medical conditions, it is possible that this factor may contribute more to prolonged opioid use than the current literature suggests; however, pharmacists should be screening patients’ medications to determine whether this risk factor may be present and follow-up with patients as necessary.^40^

Given the prevalence of statistically significance results it is likely that anxiety and depression present clinically relevant factors to take into consideration when prescribing opioids to postoperative THA and TKA patients. While the pathophysiology of anxiety and depression is complex, it has been previously shown that these conditions are associated with decreased endogenous opioid signaling, suggesting a possible mechanism for the anxiolytic effects of opioids seen in patients with anxiety/ depression.^41-43^ Because of this, it is possible that patients at the highest risk of prolonged opioid use are those with untreated anxiety/ depression or those on ineffective therapy, although no studies have been performed to determine this.

### BZD Use

Eight studies were found that reported on the risk of BZD use prior to TKA or THA.^12,13,18,21-23,28,30^ Of these, 4 reported statistically significant RRs ranging from 1.38 to 2.52.^12,13,21,22^ Because BZDs are used to treat medical conditions, such as anxiety, it follows that the RRs for BZD use are similar to those for anxiety.^44^ Since none of these studies followed RRs over time, it is hard to say how continued postoperative BZD use affects the risk of prolonged opioid use. Furthermore, it has been previously shown that BZDs may increase the risk of death 4-fold when combined with opioids due to the additive depressant effects of these medications.^45^ In 1 article, for example, BZDs have reportedly contributed to ~56% of opioid-related deaths since 2011.^46^ Finally, since BZDs are known to be a significant contributor to mortality, the use of opioids in postoperative THA and TKA patients taking BZDs should be carefully considered. If they are to be prescribed, patients should be administered the lowest effective dose of an immediate release formulation and the signs and symptoms of respiratory depression should be emphasized to try and mitigate any potential risk. As well, naloxone re-education should also be performed whenever necessary.^47,48^

### Alcohol and Substance Use

Of the 9 studies that reported preoperative alcohol or other substance use disorders (SUDs) as a risk factor for prolonged opioid use, 7 had statistically significant findings. In these studies, RRs ranged from 1.03 to 1.74 for SUDs in general^17,19,30^ and 1.1 to 2.2 for alcohol use disorders specifically.^14,21,26,27^ When measured over time, studies had conflicting results as to when the RR was at its peak. Two studies reported a peak RR at approximately 5 months postoperative,^19,26^ whereas the other 2 reported peak RR at approximately 1 year postoperative.^17,27^ In this case, it is notable that both studies reporting a peak RR at 5 months were specific to THA patients, while the 2 reporting a peak RR at 1 year were specific to TKA patients. This indicates that THA patients with a history of SUDs potentially are at a higher risk of prolonged opioid use in the short-term, whereas TKA patients with a history of SUDs are potentially at a higher risk of prolonged opioid use in the longer term. Mechanistically, there are a couple of potential reasons explaining alcohol use disorders as a risk factor. First, opioids and alcohol are known to activate similar reward pathways within the brain and do so through their overlapping analgesic and euphoric outcomes.^49^ Second, long-term alcohol use can lead to a cycle of short-term analgesia and long-term hyperalgesia, therefore, patients would be able to avoid the unwanted side effects of alcohol withdrawal by consuming opioids.^49^

In summary, 7 of 9 studies found that a history of alcohol or other SUDs was a significant risk factor for prolonged opioid use, with the risk differing between TKA and THA patients over time. Opioid prescriptions should be carefully considered in these patients due to the increased risk of sedation and respiratory depression that can occur with concomitant use of alcohol on top of the increased risk for prolonged opioid use. Risk mitigation strategies and naloxone re-education should also be performed whenever necessary.^47,48^

### Migraines

In recent years, researchers have been interested in finding new, clinically significant risk factors associated with prolonged opioid use. In our literature search, we found 4 studies examining the potential for migraines as a risk factor.^13,14,21,22^ Of these 4 papers, all of them reported statistically significant RRs ranging from 1.14 to 5.11 and 2 studies were meta-analyses.^21,22^ Notably, the meta-analysis performed by Wu et al^22^ only included 2 studies in their RR calculation for migraines, one of which was a study on shoulder arthroplasty patients and the other included solely chronic preoperative opioid patients who underwent TKA. While none of these studies provided an explanation as to why this condition is a risk factor, migraines are considered a chronic pain condition, for which opioids are occasionally prescribed to treat suggesting that it may be due to the symptomatic relief felt by patients when taking these medications.^50,51^ The current guidelines recommend that patients suffering from migraines limit their triptan, ergot, or opioid usage to no more than 9 days per month and if that is not sufficient, they should be initiated on a prophylactic medication to help reduce the need for rescue medication.^52,53^

### Fibromyalgia

The final risk factor we assessed in this review was fibromyalgia, a condition characterized by widespread musculoskeletal pain. Of the 20 studies included in this review, 7 assessed the potential for fibromyalgia as a risk factor of prolonged opioid use,^14,17,19,21,22,26,27^ and of those, 5 found statistically significant RRs ranging from 1.1 to 2.3.^14,17,21,22,27^ When measured over time, 3 studies reported the highest RR at 1 year after surgery^17,26,27^ while the other reported its highest RR at 180 to 270 days after surgery,^19^ with the trend indicating that risk increases over time. Based upon the studies that compared TKA and THA patients, there did not appear to be a difference between the RRs of these cohorts. The current guidelines state that due to a lack of evidence, opioids are generally not recommended in the treatment of fibromyalgia-related pain unless other treatments for neuropathic pain have failed; however, there may be a subset of patients with poorly controlled pain in whom opioids, such as tramadol are effective.^54,55^

## Relevance to Patient Care and Clinical Practice

One of the questions pharmacists often ask themselves when they receive a prescription for their patient is: Is this medication going to be both safe and effective for the patient? To answer that question, we need to be knowledgeable concerning the most recent literature on these topics.

While there remained some variability in the data presented here, these studies clearly indicated a number of variables can be used to predict which patient populations are at the highest risk of prolonged opioid use. By taking these risk factors into consideration, we can provide patients with more individualized health care and help reduce the burden caused by overprescribing. In the case of hip and knee replacement surgeries, this means reduced complication rates, reduced risk of adverse events, and reducing the amount of unused opioids.^6,33,35,56,57^ In addition, knowing these risk factors could potentially allow hospital pharmacists to follow-up with patients more frequently and educate those at risk of harm reduction strategies, such as naloxone use. A recent paper by VanIderstine et al^58^ goes into more depth as to the mechanism(s) underlying these risk factors.

## Conclusion

Given the prevalence of hip and knee replacement surgeries, this article aimed to discuss the recent literature surrounding risk factor for prolonged opioid use following these types of surgeries. To that end, we reported several clinically relevant risk factors which should be considered when prescribing opioids at discharge, including the age under 65, larger opioid prescriptions at discharge (> 400 MMEs), knee replacement surgery, and preoperative opioid use. This article also discussed newly established risk factors, such as migraines and fibromyalgia which may be of interest to study further. Opioid prescribing is complex and the variables many; however, we need to be using the information at our disposal to provide the best possible patient care. These risk factors should be taken into consideration for every patient and every THA/ TKA surgery, and as medication experts, pharmacists are uniquely positioned to intervene in cases where patients are receiving potentially harmful care.
